# Comparison of glucagon-like peptide-1 receptor agonists vs. placebo on any cardiovascular events in overweight or obese non-diabetic patients: a systematic review and meta-analysis

**DOI:** 10.3389/fcvm.2024.1453297

**Published:** 2024-09-11

**Authors:** Raveena Kelkar, Nishad A. Barve, Rohan Kelkar, Sanjeev Kharel, Shalmi Khanapurkar, Rukesh Yadav

**Affiliations:** ^1^ Internal Medicine, Cleveland Clinic Akron General, Akron, OH, United States; ^2^ Internal Medicine, Cleveland Clinic Mercy, Canton, OH, United States; ^3^ Department of Medicine, Bharati Vidyapeeth Deemed University Medical College, Pune, India; ^4^ Internal Medicine, Maharajgunj Medical Campus, Institute of Medicine, Tribhuvan University, Kathmandu, Nepal; ^5^ Internal Medicine, Deenanath Mangeshkar Hospital, Pune, India

**Keywords:** GLP-1 RA, obesity, cardiovascular event, non-diabetic, overweight

## Abstract

**Introduction:**

Glucagon-like peptide 1 receptor agonists (GLP-1 RA) have been extensively used to treat obesity in recent years. These novel drugs are effective at reducing body weight and also the risk of major adverse cardiovascular events in individuals with type 2 diabetes. However, the data of its efficacy in reducing cardiovascular events in individuals without type 2 diabetes is not as robust. We aim to update and conduct a systematic review and meta-analysis to assess the same.

**Methods:**

The study was conducted according to the PRISMA (Preferred Reporting Items for Systematic Reviews and Meta-Analysis) guideline. Researchers searched PubMed, EMBASE, and Clinicaltrails.gov for English literature from inception to 2024. Randomized Controlled trails enrolling adult participants (age ≥ 18 years) who are overweight or obese (BMI > 25 Kg/m^2^) with a comparison of all cardiovascular events between patients taking GLP1-RA and placebo were included. The analysis was done by Revman version 5.4.

**Results:**

A total of 17 RCTs among 34,419 participants were included in the analysis. The pooled risk ratio from 17 studies illustrated that patients with GLP-1 RA had a significantly lower risk of cardiovascular events compared to patients who had a placebo (RR = 0.75; 95% confidence interval 0.64–0.89, *p*-value = 0.0008). Semaglutide was found to have a statistically significant greatest risk reduction than other drug types.

**Conclusions:**

This meta-analysis found that GLP-1 RA significantly reduced all types of cardiovascular events in overweight and obese patients without diabetes. Semaglutide was found to be superior to others in CV event reductions. But still, the results of ongoing trials are needed.

**Systematic Review Registration:**

https://www.crd.york.ac.uk/prospero/display_record.php?RecordID=553048, PROSPERO (CRD42024553048).

## Introduction

A recent study published in the lancet found that more than a billion people worldwide are now living with obesity (1). Obesity treatment options include bariatric surgery as well as nonsurgical interventions such as nutrition change, behavioral therapy, and pharmaceutical therapy (2). Guidelines now recommend treating obesity with medications in addition to lifestyle modification (3). Glucagon-like peptide 1 receptor agonists (GLP-1 RA) delays stomach emptying, which leads to a decrease in calorie intake and encourages weight loss. It reduces appetite in the brain by directly crossing the blood-brain barrier and indirectly stimulating satiety areas through neural afferents (4). GLP-1 RA have been extensively used to treat obesity in recent years (3). These novel drugs are effective at reducing body weight and also the risk of major adverse cardiovascular events in individuals with type 2 diabetes (5). However, the data of its efficacy in reducing cardiovascular events in individuals without type 2 diabetes is not as robust. However multiple recent trials have tried to study this and the trend has been promising (6). Recent meta-analyses have shown different GLP-1 RA have reduced the risk of cardiovascular (CV) events (6, 7). We aim to update and conduct a systematic review and meta-analysis to show the risk associated with GLP-1RA utilization and the incidence of any cardiovascular events in non-diabetic individuals.

## Methods

We conducted a meta-analysis of randomized control trials (RCTs) based on PRISMA (Preferred Reporting Outcomes for Systematic Review and Meta-analysis) guidelines (8). The research question was whether GLP-1 RA decreases or increases the risk of all cardiovascular events in overweight or obese non-diabetic patients.

### Search strategy

A systematic literature search in PubMed, Embase, and ClinicalTrials.gov was done using the search strategy including MeSH terms/Emtree and keywords. The search strategy is as detailed in Supplementary File. Furthermore, we searched the reference lists of all included research and papers included in prior reviews to see whether there were any additional studies. A grey literature search was conducted using Google Scholar and Open Grey.

### Selection criteria

The criteria for inclusion of RCT studies in this review article were studies that fulfilled the following criteria:

(1) Enrolled adult participants (age ≥18 years) who were overweight or obese (BMI > 25 Kg/m^2^)
(2) Participants did not suffer from diabetes mellitus (either Type 1 or Type2)
(3) Comparison of all cardiovascular events between patients taking GLP1-RA and placebo

### Exclusion criteria

The following studies were excluded:

(1) Non-randomized studies, case reports, conference abstracts, and duplicated studies.
(2) Non-human studies

### Data extraction and quality assessments

Two independent authors (NB and RK) reviewed original articles and selected the articles as per the set eligibility criteria. Any discrepancies during the selection process were resolved through discussion with a third reviewer (RY). A data extraction spreadsheet was created on Microsoft Excel version 2013 (Microsoft Corp, Redmond, WA, USA) to extract the data under different headings; Author, Publication Year, Study Region, mean age, male/female ratio, Sample size, total dose, Follow-up durations, Cardiovascular events in both GLP-1 RA and controls. For RCT, the risk of bias tool from the Cochrane Collaboration (https://training.cochrane.org/handbook/current) was utilized as a standardized critical appraisal instrument to evaluate the risk of bias in individual studies for the primary outcome. The risk of bias was separately evaluated by two reviewers (NB and RK) based on incomplete outcome data, selective result reporting, blinding of participant personnel and outcome assessors, allocation concealment, sequence generation, and other potential sources of bias. Discussions were used to settle disagreements.

### Outcomes

The primary outcome was the occurrence of any cardiovascular event during follow-up. The secondary outcomes included (a) Major adverse cardiovascular event (MACE) defined as occurrence of death due to cardiovascular cause, non-fatal stroke or myocardial infarction, (b) Adverse cardiovascular events as reported per the Medical Dictionary for Regulatory Activities (MedDRA), (c) Cardiovascular (CV) death, (d) All-cause death, (e) Myocardial Infarction (MI), (f) Stroke, and (g) Revascularization.

### Statistical analysis

All analyses were conducted using Revman version 5.4. Pooled risk ratios (RRs) with 95% CIs were used to assess the relationship between GLP-1 RA and any CV events. The Cochrane Q-test and the *I*^2^ statistic were used to measure statistical heterogeneity. Values of *P* < 0.05 or *I*^2^ > 50% were considered significant (9). In the presence of significant heterogeneity, a random effect model (DerSimonian-Laird technique) was used (10). Otherwise, the effect was pooled using a fixed-effects model. To identify publication bias, a funnel plot, and the Eggers/Beggs test were used. Subgroup analysis was performed if necessary (11). Funnel plots were prepared and Eggers/Beggs test were done using STATA software.

## Results

### Study characteristics

**Figure 1:**
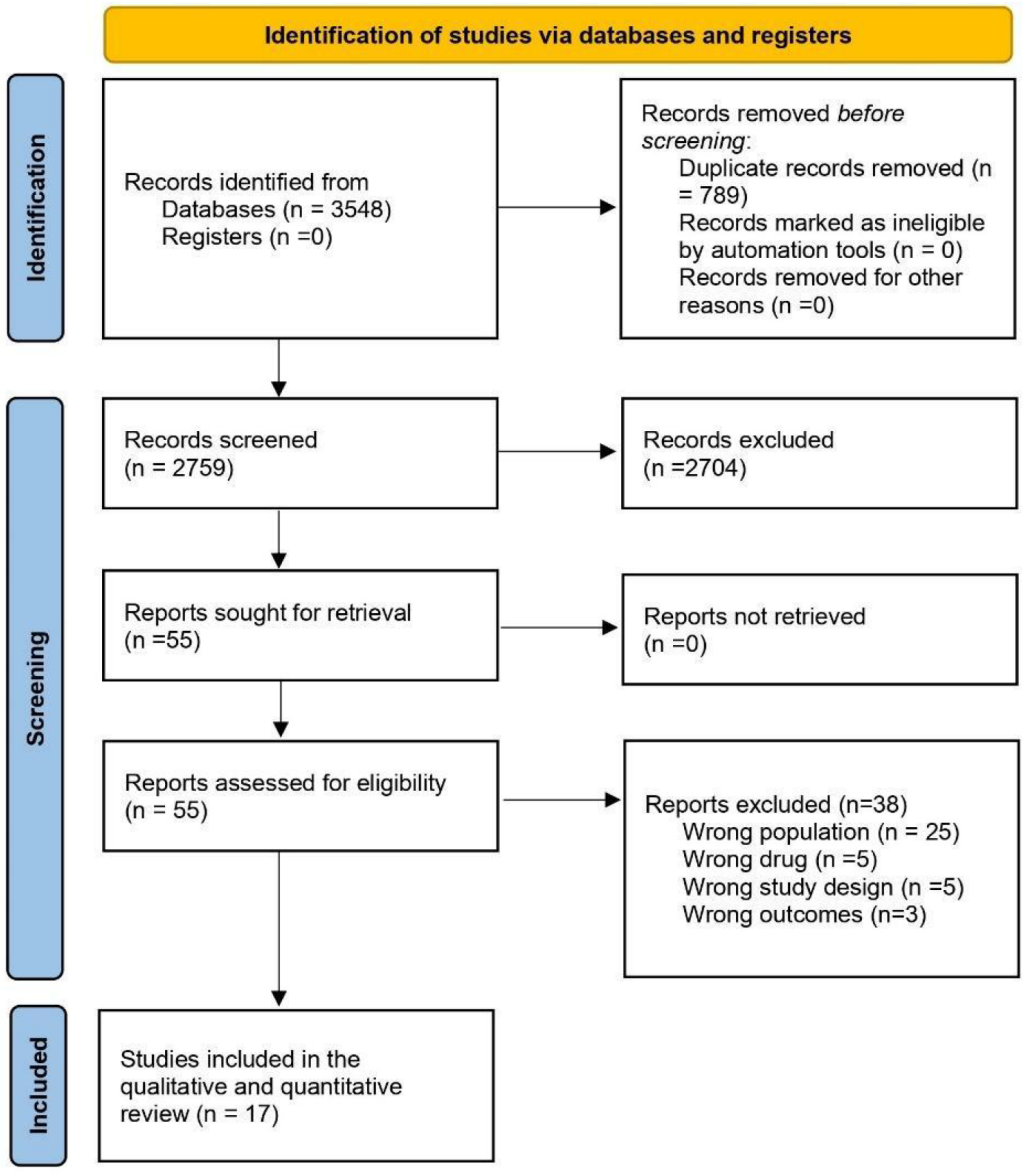
PRISMA flowchart demonstrating the details of the study selection.

A flowchart demonstrating the details of the study selection according to the PRISMA guidelines is shown in Figure 1. A total of 3,548 studies were obtained through database searches. First, we removed 789 duplicate articles and 2,759 remaining articles were screened by titles and abstracts. Furthermore, 55 articles with full text after screening were assessed as per the eligibility criteria. Finally, 17 full-text RCTs containing overweight or obese populations without diabetes treated with GLP-1 RA or placebo were included in the meta-analysis (3, 12–27). The total randomized population group ranged from 272 to 17,604. Once weekly subcutaneous semaglutide 2.4 mg was used in eight RCTs, once-daily subcutaneous liraglutide 3 mg in four RCTs, oral semaglutide in one RCT, Oral orforglipron in 1 RCT, once-weekly subcutaneous tirzepatide in 2 RCTs, 0.2 mg of beinaglutide(subcutaneous) in 1 RCT. Most of the participants in RCTs were obese females except in the SELECT trial (17) where participants majority were male. Most of the population were Caucasians. Chen et al. included Chinese patients in the trial (27). Six RCTs categorized CV events through MedDRA, 5 RCTs with MACE events while the remaining had no available categorization. The baseline demographics of the included studies are depicted in detail in Table 1.

**Table 1:** Characteristics of studies included in the meta-analysis.

Studies	Treatment group (*N*)	Placebo groups (*N*)	Intervention done	Women (%)	Mean age	Mean BMI	MACE definition	CV event type	Treatment duration weeks
STEP8 (15) 2022	253	76	Once weekly subcutaneous semaglutide 2.4 mg or once daily subcutaneous liraglutide 3 mg	265 (78%)	49	37.5	NA	MedDRA	68
STEP1 (16) 2021	1,306	655	Once weekly subcutaneous semaglutide 2.4 mg	1,453 (74%)	46	37.9	NA	MedDRA	68
STEP3 (17) 2021	407	202	Once weekly subcutaneous semaglutide 2.4 mg	495 (81%)	46	38	NA	MedDRA	68
STEP4 (18) 2021	535	268	Once weekly subcutaneous semaglutide 2.4 mg	634 (79%)	47	34.4	NA	MedDRA	48
O Neil et al.(19) 2018	821	136	Once weekly subcutaneous semaglutide (0.05 mg,0.1 mg,0.2 mg,0.3 mg or 0.4 mg) or liraglutide 3 mg	619 (65%)	47	39.3	ACS, HF, ischemic stroke, TIA, PCI	MACE	52
SELECT(20) 2023	8,803	8,801	Once-weekly subcutaneous semaglutide at a dose of 2.4 mg	4,876 (27.7%)	61.6	33.3	CV death, nonfatal MI, or nonfatal stroke	NA	39.8
SCALE Obesity and Pre-diabetes(21) 2017	1,501	747	Once daily subcutaneous liraglutide 3 mg	1,714 (75%)	47	38.9	CV death, nonfatal MI, or nonfatal stroke	MACE	160
SCALE Sleep Apnea(22) 2016	176	179	Once daily subcutaneous liraglutide 3 mg	101 (28%)	49	39.1	CV death, nonfatal MI, or nonfatal stroke	MACE	32
SCALE Obesity and Prediabetes(23) 2015	2,481	1,242	Once daily subcutaneous liraglutide 3 mg	2,928 (78%)	45	38.3	CV death, nonfatal MI, or nonfatal stroke	MACE	56
SCALE maintenance(24) 2013	212	210	Once daily subcutaneous liraglutide 3 mg	344 (82%)	46	37.9	CV death, nonfatal MI, or nonfatal stroke	MACE	56
STEP 5 (25) 2022	152	152	Once weekly subcutaneous semaglutide 2.4 mg	236 (77.6%)	47.3	38.5	NA	MedDRA	104
OASIS 1 (26) 2023	334	333	Once daily 50 mg oral semaglutide	485 (73%)	50	37.5	NA	MedDRA	68
SURMOUNT-1 (27) 2022	1,896	643	Once-weekly, subcutaneous tirzepatide (5 mg, 10 mg, or 15 mg)	1,714 (67.5%)	44.9	38	NA	NA	72
SURMOUNT-4 (28) 2024	335	335	Once-weekly, subcutaneous tirzepatide (10 mg, or 15 mg)	473 (71%)	48	30.7	NA	NA	36
STEP-HF pEF-1 (29) 2023	263	266	Once-weekly semaglutide (2.4 mg)	297 (56.1%)	69	37	NA	NA	52
Chen et al.(30) 2023	286	141	0.2 mg of beinaglutide (subcutaneous) thrice daily	218 (51.05%)	35.3	31.74	NA	NA	16
NAGZGI(31) 2023	222	50	Oral orforglipron at one of four doses (12, 24, 36, or 45 mg)	59%	54.2	37.9	NA	NA	36

MACE, major adverse cardiovascular event; MedDRA, medical dictionary for regulatory activities; NA: not available; ACS: acute coronary syndrome; HF: heart failure; TIA: transient ischemic attack; PCI: percutaneous coronary intervention; MI: myocardial infarction; CV: cardiovascular death; BMI: body mass index.

### Quality assessment results

**Figure 2:**
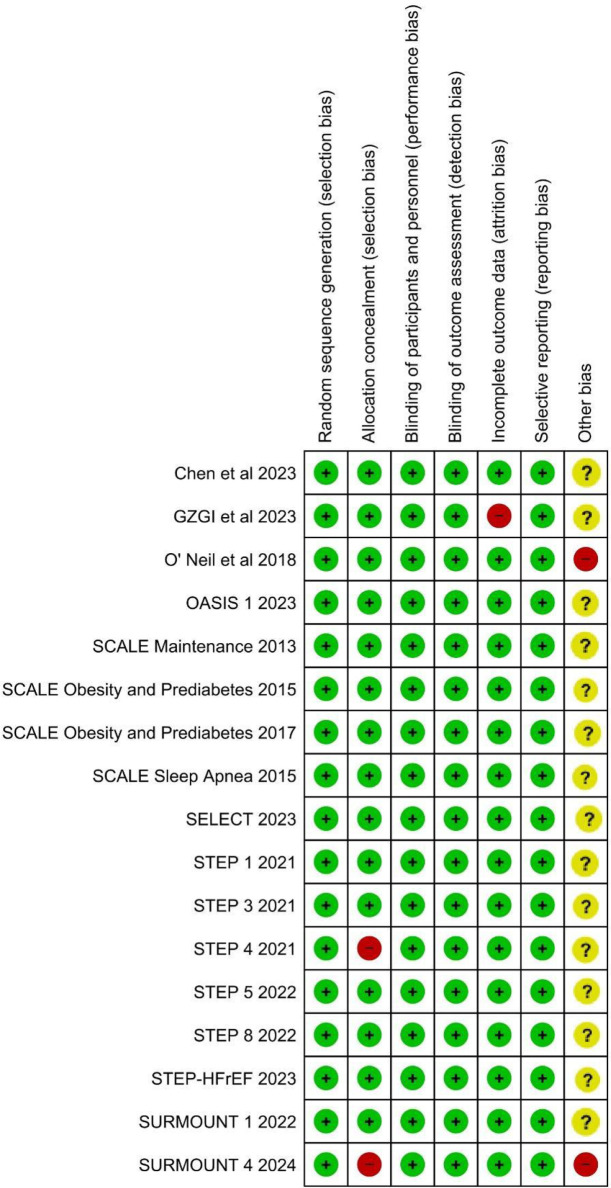
Figure showing the summary of the risk of bias among the included RCTs.

Overall, all the included RCTS had low risk of bias. Majority of trials were funded by pharmaceuticals, imparting unclear risk of bias in the “Other bias” domain of the ROB2 tool. The summary of the risk of bias is depicted in Figure 2.

## Results

**Figure 3:**
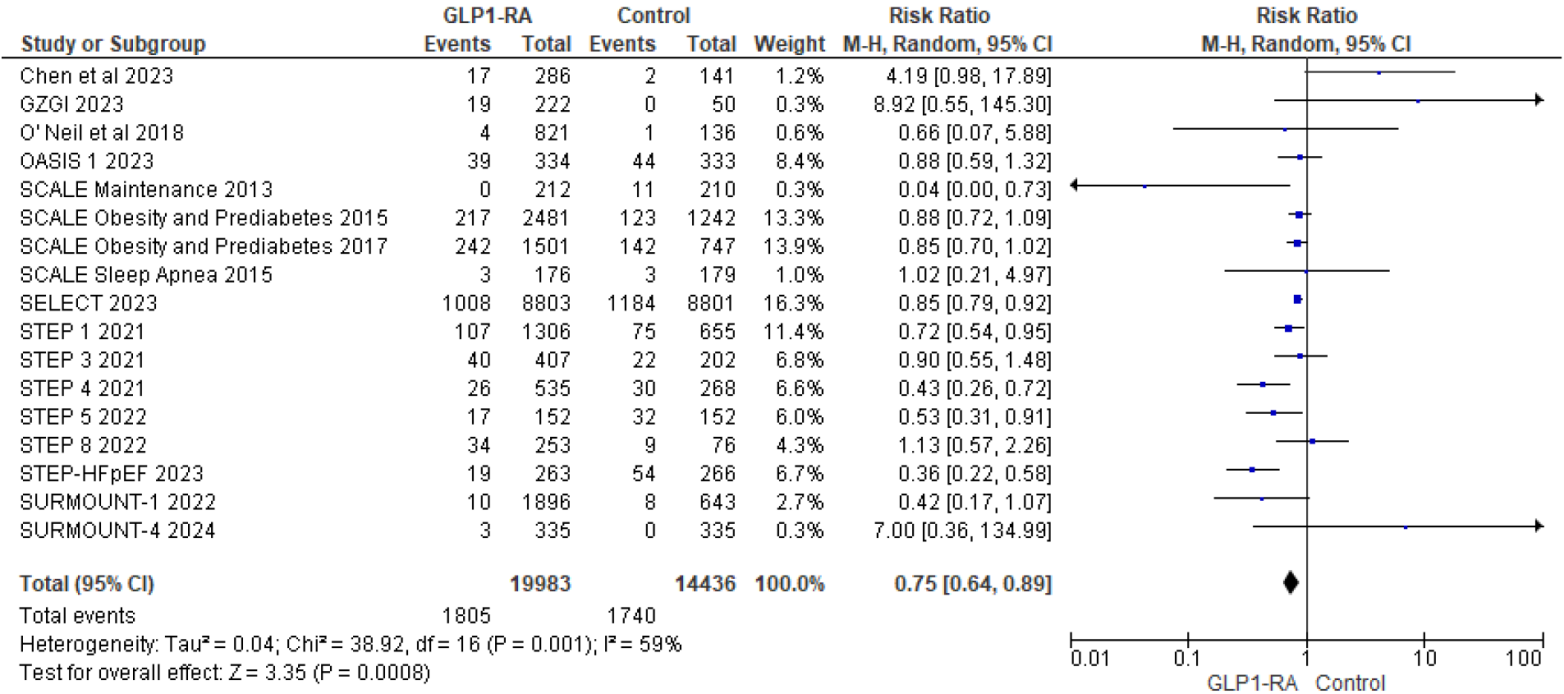
Forest plot showing the risk ratio of all cardiovascular events among GLP1-RA and placebo groups.

During follow-up, of the 19,983 participants receiving a GLP1RA, 1,805 reported an adverse cardiovascular event. Amongst the 14,436 participants receiving a placebo, 1,740 participants reported an adverse cardiovascular event. The pooled risk ratio from 17 studies (3, 12–27) illustrated that patients with GLP-1 RA had a significantly lower risk of cardiovascular events compared to patients who received a placebo (RR = 0.75; 95% confidence interval 0.64 to 0.89, *p*-value = 0.0008) (Figure 3). There was significant heterogeneity across studies (*I*^2 ^= 59%) so a random effect model was used.

**Figure 4:**
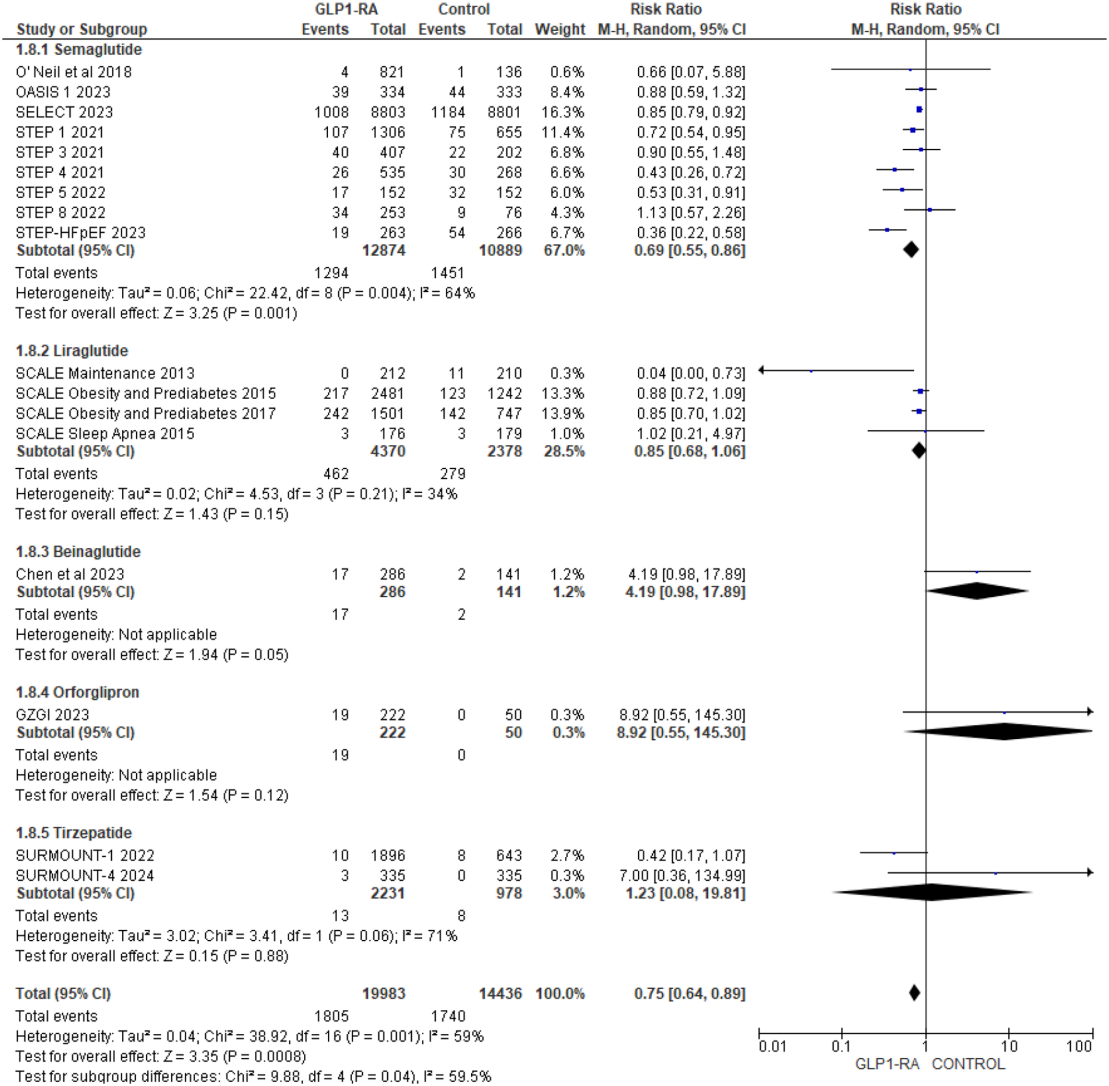
Forest plot showing the risk ratio of all cardiovascular events among GLP1-RA stratified by drug types and placebo groups.

**Figure 5:**
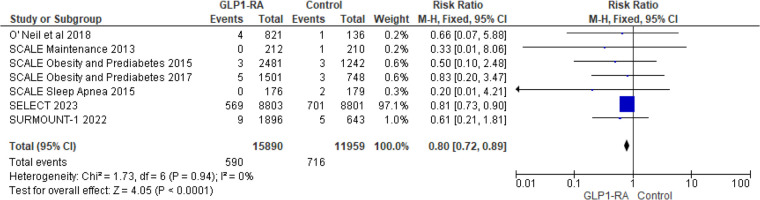
Forest plot showing the risk ratio of CV events as MACE among GLP1-RA and placebo groups.

To address heterogeneity, subgroup analysis was done according to drug type and CV events reporting type (MACE, MedDRA). Semaglutide (RR = 0.69; 95% confidence interval 0.55–0.86, *p*-value = 0.001) was found to have a statistically significant greatest risk reduction than other drug types (Figure 4). Regarding MACE events, the pooled risk ratio from five studies (16, 18–21) illustrated that patients with GLP-1 RA had a significantly lower risk of cardiovascular events compared to patients who had a placebo (RR = 0.80; 95% confidence interval 0.72–0.89, *p*-value <0.0001) (Figure 5). There was no heterogeneity across studies (*I*^2 ^= 0%) so a fixed effect model was used.

**Figure 6:**
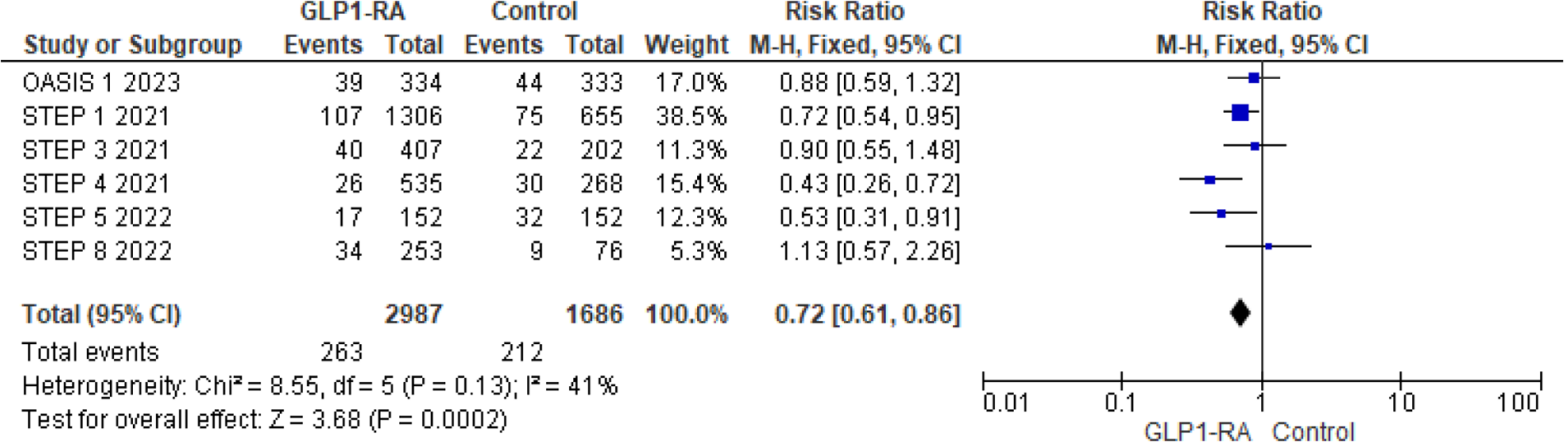
Forest plot showing the risk ratio of CV events reported as MedDRA events among GLP1-RA and placebo groups.

For MedDRA events, the pooled risk ratio from 6 studies (12–15, 22, 23) illustrated that patients with GLP-1 RA had a significantly lower risk of cardiovascular events compared to patients who had a placebo (RR = 0.72; 95% confidence interval 0.61–0.86, *p*-value = 0.0002) (Figure 6). There was minimal heterogeneity across studies (*I*^2 ^= 41%) so a fixed effect model was used.

**Figure 7:**
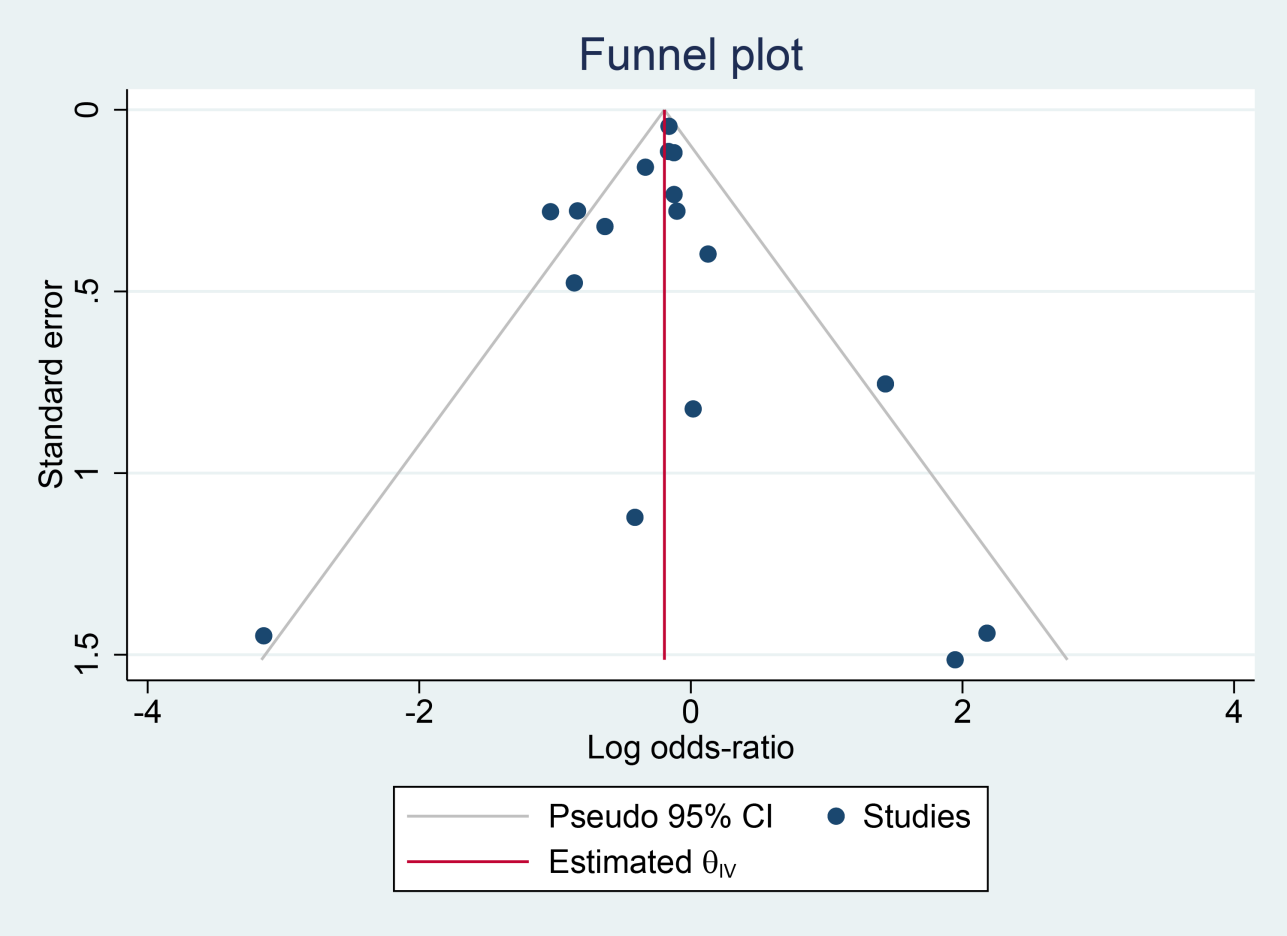
Funnel plot for publication bias detection.

**Figure 8:**
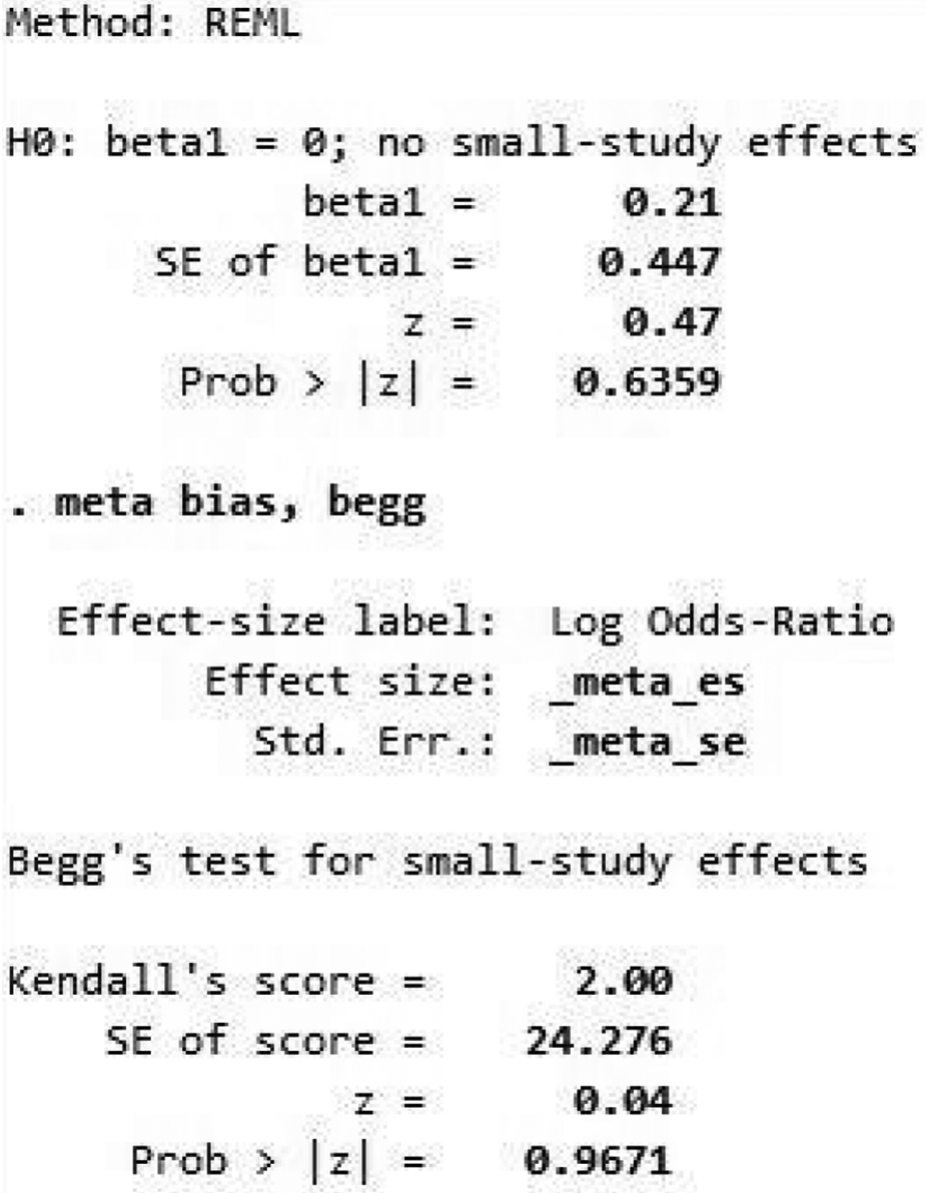
Egger and Begg's test.

Figure 7 depicts a symmetrical funnel plot, and Figure 8 shows Egger's regression test, and Begg's test for small study effects, all with *p*-values > 0.05, indicating that no significant publication bias appeared among the studies included in the analysis.

### Secondary outcomes

**Figure 9:**
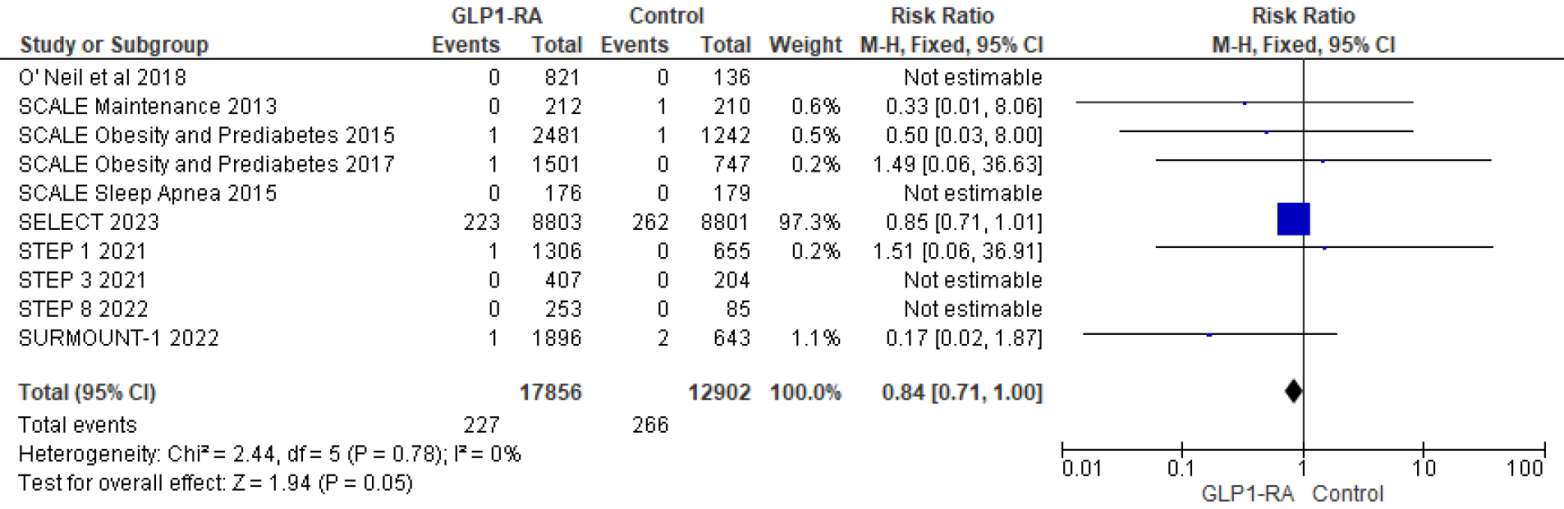
Forest plot showing the risk ratio of CV deaths among GLP1-RA and placebo groups.

**Figure 10:**
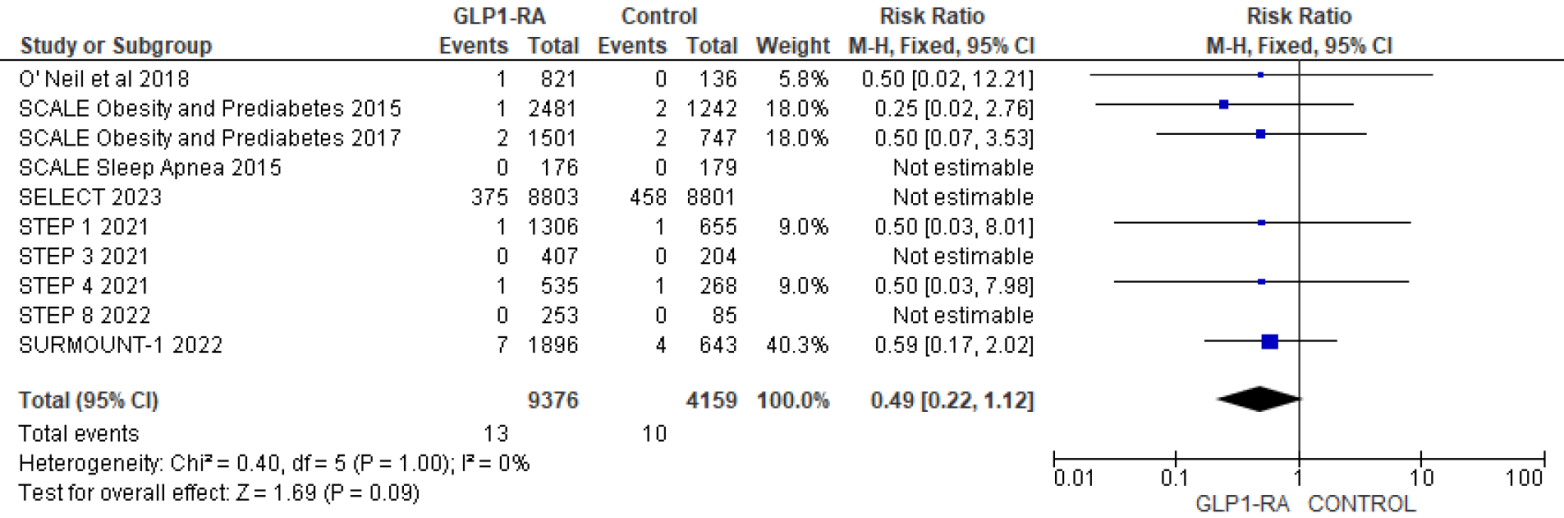
Forest plot showing the risk ratio of all cause deaths among GLP1-RA and placebo groups.

Using data from 10 studies, the analysis revealed the patients who received GLP-1 RA had near statistically significant reduction in cardiovascular death as compared to placebo (RR = 0.84; 95% confidence interval 0.71–1.00, *p*-value = 0.05, *I*^2 ^= 0%) (Figure 9). Moreover, there was non-significant reduction in all cause death with GLP-1 RA as compared to placebo (RR = 0.49; 95% confidence interval 0.22–1.12, *p*-value = 0.09, *I*^2^ = 0%) (Figure 10).

**Figure 11:**
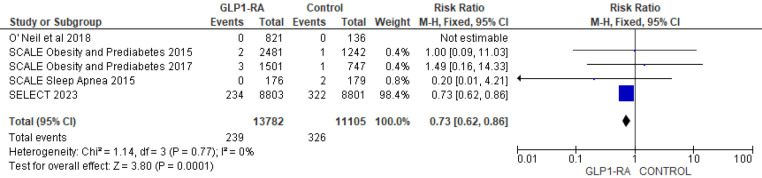
Forest plot showing the risk ratio of MI among GLP1-RA and placebo groups.

**Figure 12:**
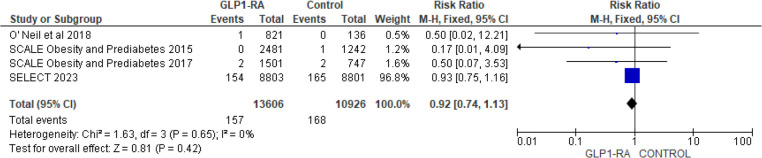
Forest plot showing the risk ratio of stroke among GLP1-RA and placebo groups.

**Figure 13:**
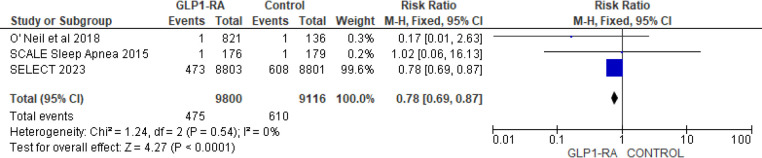
Forest plot showing the risk ratio of revascularization among GLP1-RA and placebo groups.

There was significant reduction in MI with GLP-1 RA as compared with placebo (RR = 0.73; 95% confidence interval 0.62–0.86, *p*-value = 0.0001, *I*^2 = ^0%) (Figure 11). However, the analysis revealed non-significant reduction in stroke with GLP-1 RA as compared with placebo (RR = 0.92; 95% confidence interval 0.74–1.13, *p*-value = 0.42, *I*^2^ = 0%) (Figure 12). There was significant reduction in the incidence of revascularization with GLP-1 RA as compared with placebo (RR = 0.78; 95% confidence interval 0.69 to 0.87, *p*-value <0.0001, *I*^2^ = 0%) (Figure 13).

## Discussion

The findings of this meta-analysis throw light on the potential role of GLP-1 RA drugs in non-diabetic individuals for reduction in cardiovascular risk. GLP-1 RA had a significantly lower risk of cardiovascular events compared to patients who had a placebo. Both endocrinology and cardiology societies both advised GLP-1 RAs for lowering cardiovascular risk in high-risk diabetic patients (28). According to a recent meta- analysis, GLP-1 RAs decreased the risk of MACEs by 14%, with a hazard ratio (HR) of 0.86 (95% CI, 0.80–0.93; *P* < 0.0001), all-cause mortality by 12% [HR, 0.88 (95% CI, 0.82–0.94); *P* = 0.0001], and hospital admission for heart failure by 11% [HR, 0.89 (95% CI, 0.82–0.98); *P* = 0.013] in patients with diabetes (29). The FDA also approved liraglutide (3.0 mg once daily) and semaglutide (2.4 mg once weekly) for the long-term treatment of weight in patients who are obese or overweight and have at least one weight-related comorbidity (e.g., T2D, hypertension, dyslipidemia) (30, 31). The FDA has approved tirzepatide for use in obese or overweight individuals with weight-related comorbidities (32). The use of GLP-1 receptor agonists (RAs) in patients without diabetes is still controversial, and ongoing research is investigating the therapeutic advantages and hazards of their usage (33).

To our knowledge, this is only the 3rd meta-analysis studying the cardiovascular benefit of GLP-1 RA in individuals without diabetes. The first meta-analysis by Leite et al. showed a reduction in cardiovascular events in nondiabetic participants among 11,430 patients from 9 RCTs (6). Another RCT by Singh et al. included a total of 10 RCTs with 29,325 patients (*n* = 16,900 GLP-1 RA, *n* = 12,425 placebo) and found that when compared to placebo, individuals treated with GLP-1 RAs noticed significantly lower rates of MI, revascularization, MACE, and all-cause death in overweight or obese patients without diabetes mellitus (7). We added 8 more RCTs to the 9 RCTs analyzed by Leite A. et al. and 7 more RCTs to the ones analyzed by Singh et al. and performed a meta-analysis of a total of 17 RCTs among 34,419 participants. Singh et al. used MACE, CV death, MI, Stroke and Revascularization for ascertaining CV event risk, but our study included all CV events in addition to those used by previous meta-analysis.

Though the secondary outcomes of this meta-analysis were influenced by the large trial SELECT 2023, the primary outcome (any CV events) was not affected by this trial. This highlights the potential of GLP-1 RAs in reducing the cardiovascular outcomes. SELECT 2023 trial showed that semaglutide reduces the risk of CV death or all cause death, non-fatal MI or stroke and coronary revascularization as compared to placebo. This trial randomized 17,604 non-obese and non-diabetic individuals with a follow up duration of 39.8 months. The previous SCALE, STEP and SURMOUNT group of trials did not show the reduction in the mentioned CV risks. This difference could be due to inclusion of older participants, male predominance, participants having established CV disease and CV risk factors or comorbidities in the SELECT trial. Consequently, when evaluating GLP-1 RAs for CV risk reduction in overweight/obese patients without DM, it is important to consider this particular point.

The results of this meta-analysis further strengthen the growing consensus about the cardioprotective effect of GLP-1 RA in both diabetic and non-diabetic individuals. Our study further strengthens the evidence about a potential cardio-protective effect of GLP-1 RA by utilizing a larger number of RCTs as compared to previous two meta-analyses. This study further explored the level of cardio-protection by stratifying the GLP-1 RA into individual drug groups.

### Limitations

A limitation of our study was that cardiovascular events were identified differently as reported in each RCT. A uniform definition of cardiovascular event was not utilized across the RCTs used in this meta-analysis. The major trials were limited by their predominantly female and white participants, with few data from other racial and ethnic groups. Additionally, the studies has a short follow up duration (≤72 weeks). The secondary outcomes were largely influenced by the data from the single large trial SELECT 2023.

## Conclusion

Our largest meta-analysis found that GLP-1 RA significantly reduced all types of cardiovascular events in overweight and obese patients without diabetes. Semaglutide was found to be superior to others in CV event reductions. But still, the results of ongoing trials are needed. The main challenge in future trials is the use of GLP-1 RA as a long-term solution as an oral formulation, with fewer other side effects and in different ethnic groups. As more and more research is being pursued looking into this, guidelines may soon accommodate the use of GLP1A similar to statins.

Results of the ongoing trial HISTORI (Home-based Intervention with Semaglutide Treatment Of Neuroleptica-Related Prediabetes) will shed some more light on the role of GLP-1RA in non-diabetic individuals.

## Data Availability

The original contributions presented in the study are included in the article/Supplementary Material, further inquiries can be directed to the corresponding author.

## Supplementary material

Supplementary File Supplementary search strategy.
