# Supplementary material for: Comparison of glucagon-like peptide-1 receptor agonists vs. placebo on any cardiovascular events in overweight or obese non-diabetic patients: a systematic review and meta-analysis
Source: Front Cardiovasc Med. 2024 Sep 11;11:1453297. doi: 10.3389/fcvm.2024.1453297 (PMC11422096; doi:10.3389/fcvm.2024.1453297)
Supplement: Supplementary File [file Table1.docx]

(Obesity[MeSH] OR Overweight[MeSH] OR “Weight Loss”[MeSH] OR obes*[Title/Abstract] OR "body mass ind*"[Title/Abstract] OR adiposity[Title/Abstract] OR overweight[Title/Abstract] OR "over weight"[Title/Abstract] OR "overload syndrome*"[Title/Abstract] OR overfeed*[Title/Abstract] OR "over feed*"[Title/Abstract] OR overfed[Title/Abstract] OR "over fed"[Title/Abstract] OR overfeed*[Title/Abstract] OR "over feed*"[Title/Abstract] OR overfed[Title/Abstract] OR "over fed"[Title/Abstract] OR antiobesity[Title/Abstract] OR "anti-obesity"[Title/Abstract] OR bodyweight[Title/Abstract] OR "body weight"[Title/Abstract]) AND (“Glucagon-like Peptide 1”[MeSH] OR liraglutide[Title/Abstract] OR Saxenda[Title/Abstract] OR Victoza[Title/Abstract] OR albiglutide[Title/Abstract] OR Tanzeum[Title/Abstract] OR dulaglutide[Title/Abstract] OR Trulicity[Title/Abstract] OR exenatide[Title/Abstract] OR Byetta[Title/Abstract] OR Bydureon[Title/Abstract] OR lixisenatide[Title/Abstract] OR Adlyxin[Title/Abstract]) AND ("Randomized Controlled Trial"[Publication Type] OR “randomized controlled study"[Title/Abstract] OR "randomized controlled trial"[Title/Abstract] OR "randomized study"[Title/Abstract] OR "randomized trial"[Title/Abstract] OR "randomized placebo-controlled study"[Title/Abstract] OR "randomized placebo-controlled trial"[Title/Abstract] OR "randomized placebo controlled"[Title/Abstract] OR "randomized placebo-controlled"[Title/Abstract] OR "randomized double-blin*"[Title/Abstract] OR "randomized double blin*"[Title/Abstract] OR (randomized[Title/Abstract] AND double-blin*[Title/Abstract]) OR (randomized[Title/Abstract] AND placebo-controlled[Title/Abstract]))
